# Human fetal liver cultures support multiple cell lineages that can engraft immunodeficient mice

**DOI:** 10.1098/rsob.170108

**Published:** 2017-12-13

**Authors:** Marina E. Fomin, Ashley I. Beyer, Marcus O. Muench

**Affiliations:** 1 Blood Systems Research Institute, 270 Masonic Avenue, San Francisco, CA, USA; 2 Liver Center and Department of Laboratory Medicine, University of California, San Francisco, CA, USA

**Keywords:** fetal liver, liver culture, sinusoidal endothelium, hepatocytes, haematopoietic stem cells, humanized mice

## Abstract

During prenatal development the liver is composed of multiple cell types with unique properties compared to their adult counterparts. We aimed to establish multilineage cultures of human fetal liver cells that could maintain stem cell and progenitor populations found in the developing liver. An aim of this study was to test if maturation of fetal hepatocytes in short-term cultures supported by epidermal growth factor and oncostatin M can improve their ability to engraft immunodeficient mice. Fetal liver cultures supported a mixture of albumin^+^ cytokertin-19^+^ hepatoblasts, hepatocytes, cholangiocytes, CD14^++^CD32^+^ liver sinusoidal endothelial cells (LSECs) and CD34^+^CD133^+^ haematopoietic stem cells. Transplantation of cultured cells into uPA-NOG or TK-NOG mice yielded long-term engraftment of hepatocytes, abundant LSEC engraftment and multilineage haematopoiesis. Haematopoietic engraftment included reconstitution of B-, T- and NK-lymphocytes. Colonies of polarized human hepatocytes were observed surrounded by human LSECs in contact with human CD45^+^ blood cells in the liver sinusoids. Thus, fetal liver cultures support multiple cell lineages including LSECs and haematopoietic stem cells while also promoting the ability of fetal hepatocytes to engraft adult mouse livers. Fetal liver cultures and liver-humanized mice created from these cultures can provide useful model systems to study liver development, function and disease.

## 1. Introduction

The liver is composed of a diversity of cell types including hepatocytes, cholangiocytes (biliary epithelial cells), different types of endothelial cells and multiple lineages of blood cells. All of these cell types, and in particular their precursors, are of great interest to the field of regenerative medicine. The liver has an extensive capacity for regeneration and understanding the mechanisms that control liver growth may one day lead to new cell therapies or bioengineered livers [1,2]. Pluripotent stem cells offer a new source of cells for such novel therapies that does not further constrain the already limited supply of donor liver tissue [3]. However, use of pluripotent stem cells to generate liver cells requires an understanding of liver development if these stem cells are to be used effectively. Baxter *et al*. [4] recently demonstrated that hepatocyte-like cells derived from embryonic stem cells have a molecular signature more like fetal cells than adult hepatocytes. Accordingly, greater knowledge of the early development of the human liver will likely inform efforts to use pluripotent stem cells to create new liver tissue.

There are numerous differences, ranging from gross cell composition to gene expression of individual cell lineages that distinguish the adult and fetal liver. During prenatal development, the two major cell lineages that constitute the adult liver, hepatocytes and cholangiocytes, are found in the presence of an abundance of haematopoietic cells not observed in adult tissue. Haematopoiesis is first observed in the human embryonic liver at five weeks' gestation [5,6]. Haematopoietic stem cells that seed the embryonic liver are derived from the haemogenic endothelium of the dorsal aorta [7]. The liver, in turn, is the major haematopoietic organ in the human embryo and fetus until midway through gestation. The onset of bone marrow haematopoiesis begins around the 13th week of gestation [8], and not until about 20 weeks' gestation does the content of CD34^+^ haematopoietic progenitors in the bone marrow reach parity with the liver [9,10]. The midgestation liver supports multilineage haematopoiesis [11–13]; however, erythropoiesis greatly exceeds all other types of blood cell production [14,15]. By one estimate, CD235a^+^ erythroid cells comprise the majority of all cell types in the midgestation liver [16].

There has been only limited study of the haematopoietic niches within the developing human liver. Erythroid precursors have been found among fetal hepatocytes and within liver sinusoids [17], whereas myeloid precursors were observed in association with vascular structures of the portal triads [18]. No specific histological niche for human B-lymphopoiesis has been found as B-cell progenitors appeared scattered throughout the liver parenchyma [19]. Haematopoietic stem cells, in the mouse embryonic liver, have been found associated with pericytes surrounding portal vessels [20]. The niches for human haematopoietic progenitors and stem cells have not yet been defined.

The fetal liver contains a number of endothelial cell types that line the large vessels of the liver, the portal veins and hepatic artery, as well as the endothelial cells that line the lymphatic vessels. The majority of the endothelial cells found in the liver, however, are liver sinusoidal endothelial cells (LSECs) [21]. These cells have some unique properties, including being the major source of factor VIII, and transplantation of LSECs has been proposed as a therapy for treating haemophilia A [22]. The molecular signals that support the development of LSECs in the liver are largely unknown, but a further understanding of the requirements of these cells will be needed to design culture systems to expand LSECs or generate them from stem cells.

Numerous differences between fetal and adult hepatocytes have been documented [23–25]. The human fetal liver is rich in hepatoblasts, hepatocytic precursors that can differentiate into hepatocytes and cholangiocytes [16,26]. Hepatoblasts can be identified by their co-expression of the hepatocyte marker albumin and the cholangiocyte marker cytokeratin (CK)19 [27]. These bi-potent cells are found in the fetal liver surrounding ductal plates [25,28]. The surface-marker epithelial cell adhesion molecule, or CD326, is expressed by hepatoblasts as well as the immature hepatocytes found in the parenchyma of the fetal liver. By contrast, adult hepatocytes do not express CD326, which is only found on a rare subset of adult hepatic stem cells [25,29]. Whereas fetal cells, in general, are characterized by a high proliferative activity *in vitro* and *in vivo*, there have been reports of little or no engraftment of fetal hepatocytes/hepatoblasts transplanted into immunodeficient mice compared with adult hepatocytes [29,30]. These findings suggest that the unique properties of immature fetal hepatocytes and their precursors render the cells less suited to engraftment and/or growth in the adult liver.

Our first aim in this study was to test the hypothesis that maturation of human fetal hepatocytes in short-term cultures would improve engraftment in adult mice. Additionally, we sought to determine if fetal liver cultures could support multiple cell lineages found in the fetal liver other than hepatocytic cells, namely LSECs and haematopoietic cells. Lastly, we tested if transplantation of cultured fetal liver cells could not only lead to hepatocyte engraftment but also engraftment of LSECs and haematopoietic stem cell engraftment.

## 2. Material and methods

### 2.1. Procurement and processing of human liver tissue

Human fetal livers were obtained from elective legal abortions with the written informed consent of the women undergoing the procedure and the approval of the Institutional Review Board at the University of California San Francisco (IRB# 10-00768). Specimens were donated anonymously at San Francisco General Hospital. This research was conducted in accordance with the Declaration of Helsinki. The gestational age of the specimens was estimated based on foot-length measurements. Tissues were obtained shortly after abortion and held on ice in cold phosphate-buffered saline (PBS) and antibiotics for delivery to the laboratory within 4 h.

Livers were manually disrupted with scissors and digested with 0.005% DNAse and a blend of Collagenase I/II and Thermolysin (Roche Diagnostic Corporation, Indianapolis, IN) for 15 min at 37°C. The reaction was then stopped with cold fetal bovine serum (FBS). Cells were then washed with PBS and CD235a^+^ red cells were depleted using BioMag goat anti-mouse IgG immunomagnetic beads (Qiagen Inc., Germantown, MD) as previously described [31]. CD235a-depleted cells were used for all experiments.

Cryopreserved human adult hepatocytes (Cat#HMCPTSA and Cat#HMCS10) were purchased from Life Technologies (Carslbad, CA).

### 2.2. Tissue culture

CD235a^−^ liver cells were plated in William's E Medium (Thermo Fisher Scientific, Grand Island, NY) with 5% FBS and Primary Hepatocyte Maintenance Supplements (#CM4000, Thermo Fisher Scientific) providing 0.1 µM dexamethasone, 6.25 µg ml^−1^ human recombinant insulin, 6.25 µg ml^−1^ human transferrin, 6.25 ng ml^−1^ selenous acid, 1.25 mg ml^−1^ bovine serum albumin, 5.35 µg ml^−1^ linoleic acid, 2 mM GlutaMAX and 15 mM 4-(2-hydroxyethyl)-1-piperazineethanesulfonic acid. Cultures were also supplemented with 10 ng ml^−1^ oncostatin M (OSM) and 10 ng ml^−1^ epidermal growth factor (EGF). Cells were grown on BioCoat Collagen I coated tissue culture plates (BD, Franklin Lakes, NJ) for 6 days at 37**°**C. Confluent cultures were harvested using TrypLE Enzyme solution (Life Technologies) and then stained for flow cytometric analysis or transplantation into mice. Some plates were fixed with 10% formalin in PBS and stained for immunofluorescence microscopy analysis.

### 2.3. Xenogeneic mouse transplants

Animal transplants were conducted with approval of the Institutional Animal Care and Use Committee at PMI Pre-clinical (San Carlos, CA). The reported research was performed under protocol numbers IAC 1361 and IAC 1745. All animals were bred and maintained in our specific-pathogen free animal facility. NOD.Cg-Prkdc^scid^ Il2rg^tm1Sug^ Tg(Alb-Plau)11-4/ShiJic (uPA-NOG) mice were bred as a cross of homozygous males with hemizygous females [32]. Offspring were phenotyped based on plasma levels of serum alanine transaminase (ALT) to determine genotype [33]. Male and female animals with ALT levels higher than 100 units were considered homozygous and were used for transplantation.

NOD.Cg-*Prkdc^scid^ Il2rg^tm1Sug^ Tg(Alb-UL23)7-2*/ShiJic (TK-NOG) mice were bred as crosses of wild-type NOG males and hemizygous TK-NOG females. Breeding pairs of TK-NOG and uPA-NOG mice were kindly provided by Dr Hiroshi Suemizu of the Central Institute for Experimental Animals (Kawasaki, Japan). TK-NOG offspring were genotyped with Kapa genotyping kit (Kapa Biosystems, Wilmington, MA) as per the manufacturer's instructions using primers as previously described [34]. Hemizygous male and female mice, 6–8 weeks old, were injected with Ganciclovir (EDM Millipore Corp., Darmstadt, Germany) 7 and 5 days before transplantation to induce liver damage. Animals were transplanted by intrasplenic injection and sacrificed using CO_2_ followed by cervical dislocation at different time points post transplant (130–150 days).

### 2.4. Analysis of mice for engraftment of human cells

Levels of human serum albumin in transplanted mice were determined as previously described [33].

Livers, spleens and pancreases were harvested and fixed with 10% formalin for sectioning and immunofluorescence staining. Tissues were processed through a sucrose gradient (10%, 20%, 30% and 50% in PBS) and frozen in Tissue-Tek Optimal Cutting Temperature compound (Ted Pella Inc., Redding, CA). Cryo-blocks were sectioned on a Cryostat Leica CM1850 UV (Leica Biosystems Nussloch GmbH, Nussloch, Germany) and stained with primary and secondary antibodies as previously described [30].

Flow cytometry was used to analyse the engraftment of livers and spleens by human cells as previously described [30,33]. Livers and spleens were digested with Life Technologies' Collagenase IV (Thermo Fisher Scientific). The light-density fractions of spleen cells were collected by centrifugation over a layer of Axis-Shield's Lymphoprep (STEMCELL Technologies Inc., Vancouver, BC). Flow cytometry was also used to analyse engraftment of human haematopoiesis in the central bone marrow, which was collected as previously described [35]. Staining with fluorescent antibodies was performed as described [28,30].

### 2.5. Data analysis and presentation

Flow cytometry data were analysed using FlowJo software, v. 9 (FlowJo, Inc.; Ashland, OR). Statistical analysis and charting was performed using Aabel 3 and Aabel NG software (Gigawiz Ltd. Co. OK, USA). The non-parametric Mann–Whitney U test was used to compare the frequencies of cell populations and engraftment. A *p*-value of ≤0.05 was considered significant. Albumin concentrations from multiple mice are presented in the text as the mean ± s.e. Some data are presented using notched box and whisker plots in which the notch corresponds to the median and the box extends from the 25th to the 75th percentiles. Whiskers extend to the extreme data points. Means are also indicated using a flattened diamond symbol. Individual data points, when shown, are indicated as open equilateral diamonds.

## 3. Results

### 3.1. Establishment of multilineage human fetal liver cultures

Different culture media have been developed to support the *in vitro* growth and survival of various types of fetal liver cells. For example, we have successfully used commercially available endothelial cell growth medium to grow LSECs [30]. Haematopoietic precursors of multiple lineages can be maintained in defined media formulations based on Iscove's Modified Dulbecco's Medium and purified serum components [9,31,36], and culture medium based on Williams's E medium [37] as described by Lázaro *et al*. [38] can be used to successfully grow fetal hepatocytes. *In situ*, all these cell types develop and are maintained together with numerous cell–cell interactions playing a role in maintaining tissue homeostasis. We aimed to model this interaction among hepatocytic, endothelial and haematopoietic cells *in vitro* in cultures using Williams's E medium, containing supplements used for hepatocyte growth and the cytokines OSM and EGF. These conditions have already been shown to be sufficient to support fetal CD326^+^ hepatoblasts [28].

**Figure 1.**
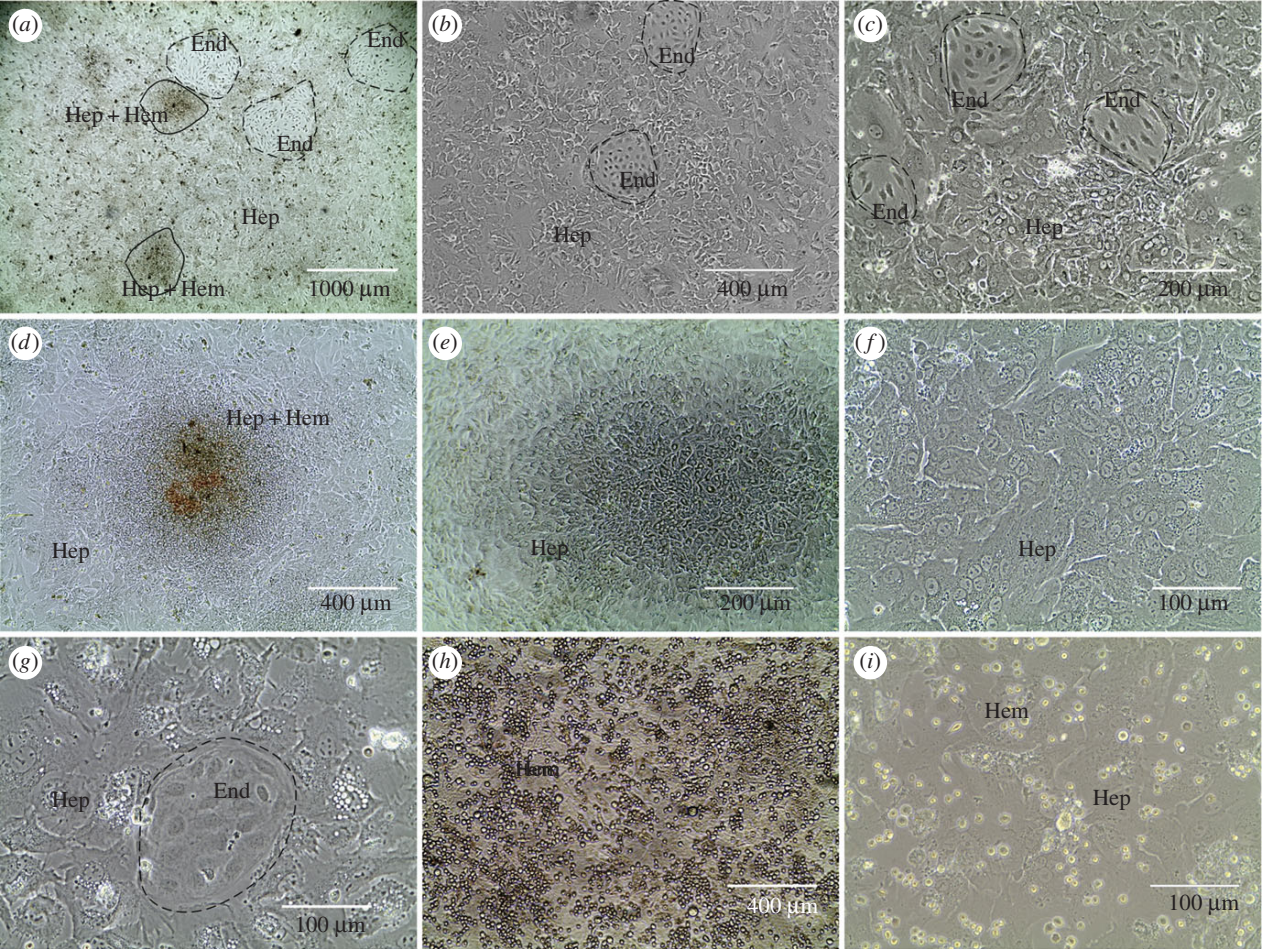
Phase-contrast images of fetal liver cells cultured for 6 days. (*a*) Hepatocytes form adherent layer of cells with islands of endothelial cells (larger on *b*, *c* and *g*), and dark tight colonies of cells, representing small hepatocytes (*e*) often with haematopoietic cells attached (*d*). Non-adherent haematopoietic cells are also present in the culture (round cells on *h* and *i*). (*f*) Close-up image of hepatocytes. Hep, hepatocytes; Hem, haematopoietic cells; End, endothelial cells.

Erythrocyte-depleted fetal liver cells were cultured and, after 5–6 days, three prominent types of cells were observed by phase-contrast microscopy (figure 1). Most adherent cells appeared to be hepatocytes (figure 1), with islands of apparent endothelial cells (figure 1*a*,*b*,*c*,*g*), and dispersed haematopoietic cells (figure 1*h*,*i*). Two hepatocyte morphologies were observed. Some of them were large as in figure 1*c*,*f*,*g*, polygonal and multinucleated. Others were small and tightly packed, as in figure 1*e*, forming colony-like structures, which were often associated with haematopoietic cells (figure 1*a*,*d*). Endothelial cells formed clusters with a typical cobblestone morphology.

**Figure 2.**
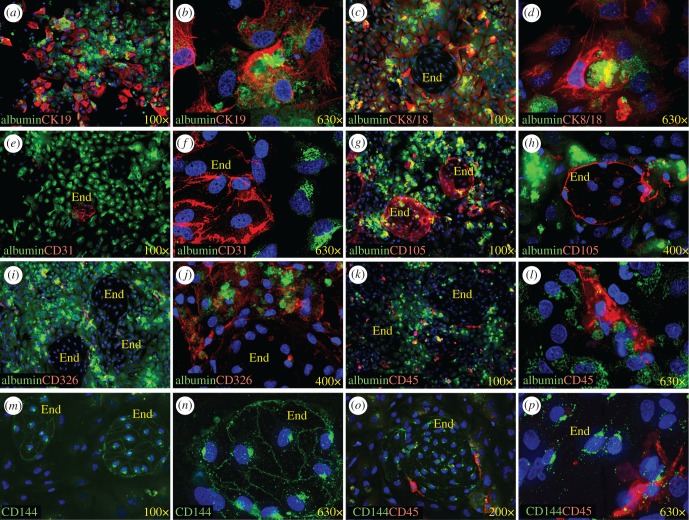
Immunofluorescence staining reveals a diversity of cell types in fetal liver cultures. Most of cells are hepatocytes expressing albumin (all panels but *m*–*p*). They coexpress CK19 (*a*,*b*), CK18 (*c*,*d*) and CD326 (*i*,*j*). Endothelial cell islands express CD31 (*e*,*f*), CD105 (*g*,*h*) and CD144 (*m*–*p*). CD45^+^ haematopoietic cells are found among hepatocytes but are also concentrated around endothelial cells (*k*,*l*). Nuclei were stained with 40,6-diamidino-2-phenylindole (DAPI; blue, all panels). End, endothelial cells.

**Figure 3.**
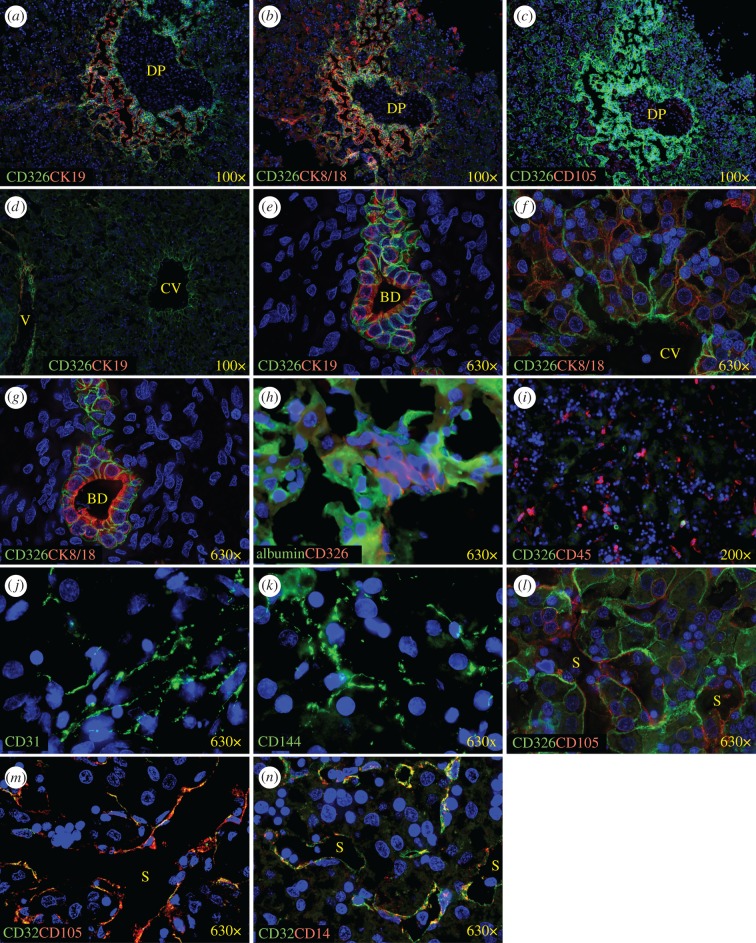
Immunofluorescence staining of human fetal liver. Fetal liver sections were stained with the indicated fluorochrome-conjugated antibodies. The gestational ages of the specimens analysed were 9.5 weeks (*a*–*c*) and 24 weeks (*d*–*n*). Nuclei were stained with DAPI (blue). DP, ductal plate; CV, central vein; V, vessel; BD, bile duct; S, sinusoid.

Immunofluorescent staining confirmed the presence of different cell types in the fetal liver cultures (figure 2). Cells forming the main adherent layer expressed the hepatocyte marker albumin (figure 2*a*–*l*), the cholangiocyte/hepatoblast marker CK19 (figure 2*a*,*b*) as well as CK8/18, which is expressed by both lineages (figure 2*c*,*d*). For comparison, sections of fetal liver were similarly stained as a point of reference for normal tissue architecture (figure 3). CK19 expression was evident around ductal plate structures in first trimester liver (figure 3*a*) and in the perimeter of some vascular structures of midgestation liver (figure 3*d*). CK8/18 was expressed in a similar pattern as CK19, but was also widely expressed by parenchymal cells (figure 3*b*,*f*). Expression of CK19 and CK8/18 was also evident on cholangiocytes forming the bile ducts observed within the mesenchymal connective tissue (unstained cells) of portal tracts (figure 3*e*,*g*). Among cultured cells, it was evident that some of the cultured cells express more albumin than CK19 or CK8/18, whereas others express more of these CKs (figure 2*a*,*c*), which points to differentiation towards the hepatocyte and cholangiocytes lineages, respectively. However, at higher magnification it appears that most hepatocytic cells coexpress albumin and the CKs markers to some degree (figure 2*b*,*d*), suggesting the presence of hepatoblasts and cells at intermediate stages of differentiation.

CD326 is a marker that is observed on fetal but not on adult hepatocytes [24]. CD326 highlights hepatoblasts throughout the parenchyma of fetal livers (figure 3*a*–*i*,*l*) with more intense staining in ductal plate regions (figure 3*a*–*c*) and on the cells surrounding vessels observed in midgestation tissues (figure 3*d*,*f*). CD326 was also expressed on cholangiocytes (figure 3*e*,*g*) and was co-expressed with albumin in the parenchyma of the fetal liver (figure 3*h*). Likewise, both CD326 and albumin expression were widely observed on cultured fetal liver cells (figure 2*i*,*j*).

Endothelial cell islands within the fetal liver cultures were negative for albumin and other hepatocytic markers (figure 2*c*,*i*–*k*), but did express the endothelial markers CD31 (figure 2*e*,*f*), CD105 (figure 2*g*,*h*) and CD144 (figure 2*m*–*p*). These same markers, as well as CD32, were also expressed by endothelial cells lining sinusoids in the fetal liver (figure 3*j*–*n*).

Haematopoietic cells, identified by CD45 expression, are found widely dispersed throughout the parenchyma of the fetal liver (figure 3*i*). In the staining of the cultured cells, lightly adherent cells or non-adherent cells were lost during the wash and staining procedures and, therefore, were not represented in these analyses. Thus, haematopoietic cells represented in the stained samples were adherent cells mostly localized around endothelial cells (figure 2*k*,*l*,*o*,*p*).

**Figure 4.**
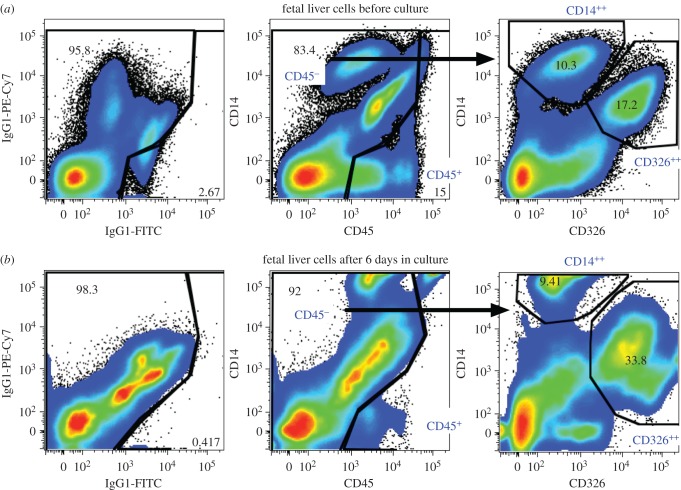
(*a*,*b*) Flow cytometric analysis of fetal liver cells before and after culture. Live cells lacking propidium iodide staining were gated and the numbers in all panels represent the percentage of live cells. Background staining with isotype-control antibodies reveal the complexity of cell types in the culture as seen by the extensive spread in background signals. Haematopoietic and non-haematopoietic cells were distinguished based on CD45 expression. Among non-haematopoietic cells two subpopulations were selected for further analysis: CD14^++^ cells and CD326^++^ cells.

We previously described a panel of cell-surface markers useful for flow cytometric discrimination of major cell lineages in the fetal liver using CD14, CD45 and CD326 [28,30]. Among freshly isolated cells (figure 4*a*), CD45 expression identifies nucleated haematopoietic cells; low expression of CD14 and high expression of CD326 on cells lacking CD45 expression marks hepatoblasts (referred to as CD326^++^ cells); additionally, bright CD14 expression on CD45^−^ cells identifies LSECs (referred to as CD14^++^ cells). Here we show that the same cells populations present in fresh fetal liver can be found after short-term culture (figure 4*b*). In three experiments, the proportion of LSECs among all live cells after culture was a mean 6.4%, whereas CD326^++^ cells represented a mean 26.4% of cultured cells. This is consistent with the proliferation of hepatocytic cells under culture conditions that favour hepatocyte growth, but is also probably affected by the decrease in CD14^−^CD326^−^ cells believed to be non-adherent cells that are depleted due to washing of the cultures. Despite the loss of some non-adherent cells, a mean of 10.4% CD45^+^ cells were observed among the live cells harvested from the cultures.

**Figure 5.**
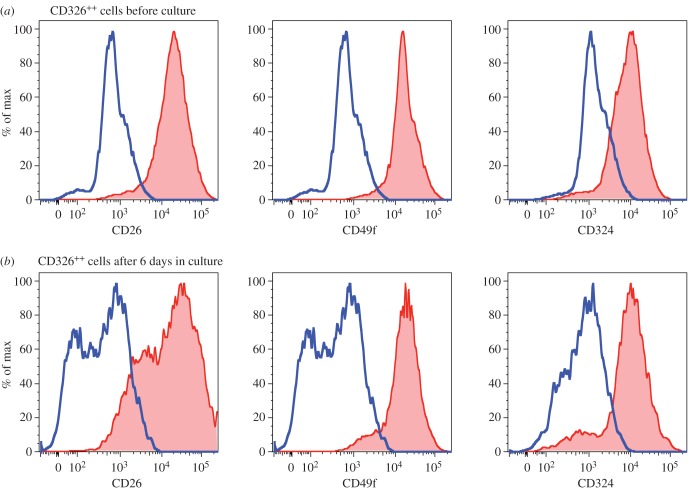
(*a*,*b*) CD326^++^ cells after culture continue to express hepatocyte markers CD26, CD49f and CD324. CD326^++^ cells were defined by gating on CD45^−^CD326^++^CD14^low^ cells as indicated in figure 4. On the histograms, blue represents the isotype control and red corresponds to specific staining with the indicated antibody.

**Figure 6.**
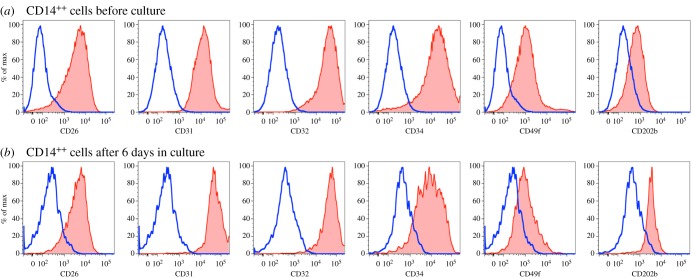
(*a*,*b*) Expressional profile of CD14^++^ cells before and after culture as demonstrated by flow cytometric analyses. CD14^++^ cells were defined by gating on CD45^−^CD14^++^ cells as indicated in figure 4. On the histograms, blue is the isotype control and red represents staining with the indicated antibody.

Additional lineage markers were evaluated before and after culture to verify the presence of multiple cell lineages among the cultured cells. Detailed analysis of the CD326^++^ cell population demonstrated that expression of CD26, CD49f and CD324, found on fresh hepatoblasts [28], was preserved after culture (figure 5). Analysis of CD14^++^ cells showed the expression of CD26, CD31, CD32, CD34, CD49f and CD202b before (figure 6*a*) and after (figure 6*b*) culture, consistent with the known phenotype of fetal LSECs [30].

**Figure 7.**
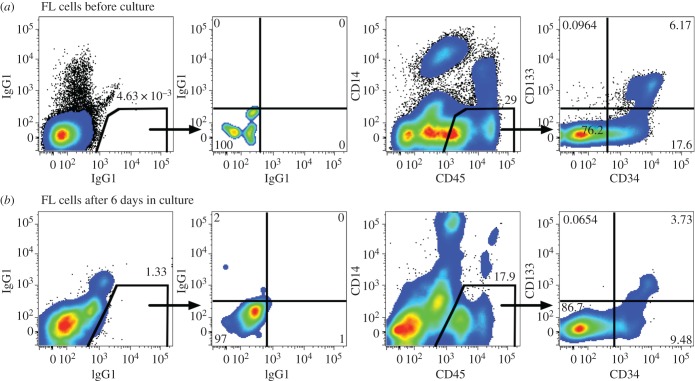
(*a*,*b*) Haematopoietic cells in the fetal liver before and after culture. Gates were set on small-live cells (not shown) to enrich for CD34^+^ haematopoietic precursors shown in the figure.

The fetal liver is a rich source of haematopoietic stem cells, which have a phenotype CD45^+^CD34^+^CD133^+^ [39], as shown in figure 7*a*. Cells with this phenotype were also observed among the cultured cells (figure 7*b*). Moreover, a spectrum of CD34 expression was observed, suggesting the presence of haematopoietic progenitors at different stages of differentiation and maturation. A population of CD14^+^CD45^+^ cells was also observed, probably representing monocyte/macrophages. Thus, the analyses of cells from the fetal liver cultures confirmed that at least three cell lineages (hepatocytic, endothelial and haematopoietic) were present in the cultures after 6 days of growth under conditions that favour the growth of hepatocytic cells.

### 3.2. Human engraftment in mice

The uPA-NOG strain of mice can be engrafted with adult human hepatocytes [32,33,40], but we previously failed to observe engraftment when these mice were transplanted with fresh fetal liver cells [30]. Table 1 summarizes data from seven experiments in which erythrocyte-depleted fetal liver cells were transplanted into uPA-NOG mice. All the mice were engrafted with human cells based on flow cytometric analyses. Only one mouse, however, yielded evidence of hepatocyte engraftment based the analysis of human albumin at 10 ng ml^−1^ in the serum, and no cells expressing albumin were detected by immunofluorescence staining of liver sections. The human cells present in the livers of these mice were comprised of LSECs and haematopoietic cells as previously reported [30].

**Table 1.** Liver engraftment in uPA-NOG mice transplanted with fresh fetal liver cells.

experiment	days in culture	no. cells transplanted	time to harvest (days)	no. mice analysed	number engrafted with human cells	number engrafted with human hepatocytes
1	0	5 × 10^5^	100	6	6	1
2	0	1 × 10^6^	130	8	8	0
3	0	1 × 10^6^	130	8	8	0
4	0	1 × 10^6^	130	5	5	0
5	0	1 × 10^6^	203	2	2	0
6	0	1.5 × 10^6^	189	2	2	0
7	0	3 × 10^6^	201	3	3	0
8	6	8 × 10^5^	43–155	8	8	4
9	6	5 × 10^5^	138–160	10	10	7
10	6	3–8 × 10^5^	169–175	7	5	2

**Figure 8.**
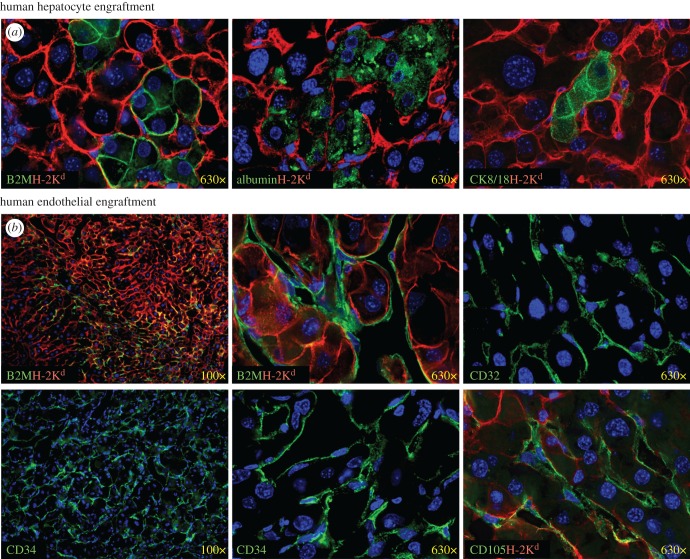
Immunofluorescent staining of human engraftment in mouse liver sections. Hepatic engraftment (*a*) and LSEC engraftment (*b*) defined by morphology and tissue specific human markers (green). Murine cells are stained with pan-mouse marker H-2K^d^ (red). Nuclei are stained with DAPI (blue).

We tested if hepatocytes derived from cultured human fetal liver cells could engraft uPA-NOG mice (table 1). Evidence of engraftment of mouse livers by human hepatocytes, LSECs and haematopoietic cells was observed in three independent experiments. Clusters of human hepatocytes were detected in the murine liver by expression of the pan-human marker β-2 microglobulin (B2M) and the human hepatocyte-markers albumin and CK8/18 (figure 8*a*). Significantly more mice were found to be engrafted with hepatocytes when transplanted with cultured cells than with fresh fetal liver cells based on the analysis of the frequency of mice detected in which hepatocytes were observed in the livers and/or human albumin was detected in the serum (*p* = 0.0167). Human albumin was detected in the serum of mice in experiments 9 and 10 at 16.2 ± 10.1 µg ml^−1^ and 0.39 ± 0.14 µg ml^−1^, respectively. Human LSECs, expressing B2M, were morphologically different from hepatocytes and were found dispersed between mouse hepatocyte populations, as previously observed [30]. These LSECs expressed the endothelial markers CD32, CD34 and CD105 (figure 8*b*).

**Figure 9.**
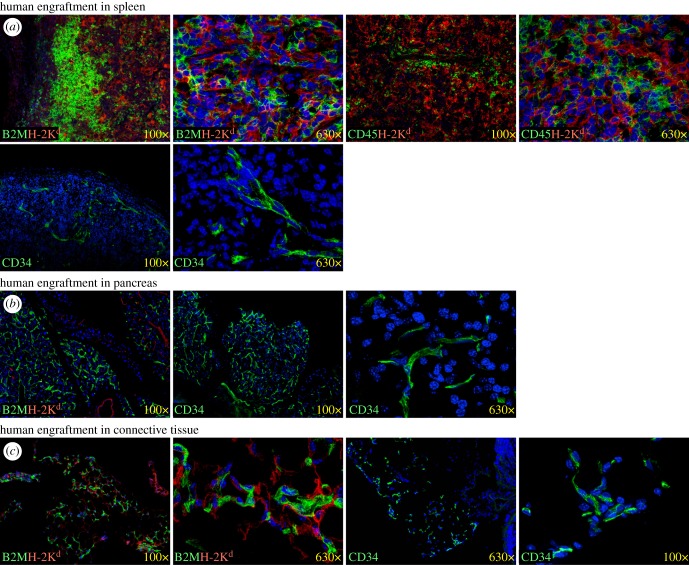
Human engraftment in extra-hepatic tissues. (*a*) Haematopoietic and endothelial engraftment in the spleen. (*b*) Endothelial engraftment in the pancreas. (*c*) Endothelial engraftment in connective tissues associated with the spleen. Human markers are shown in green, mouse H-2K^d^ is shown in red and DAPI-stained nuclei are blue.

Spleens were engrafted mostly with small rounded CD45^+^ haematopoietic cells, but also some elongated CD34^+^ endothelial cells were detected (figure 9*a*). Human endothelial cells were also detected in mouse pancreases (figure 9*b*) and connective tissues associated with the spleen (figure 9*c*). CD32, a specific marker for LSECs was not detected on human endothelial cells in extra-hepatic tissues (data not shown), suggesting a loss or change in the LSEC phenotype outside the liver.

**Figure 10.**
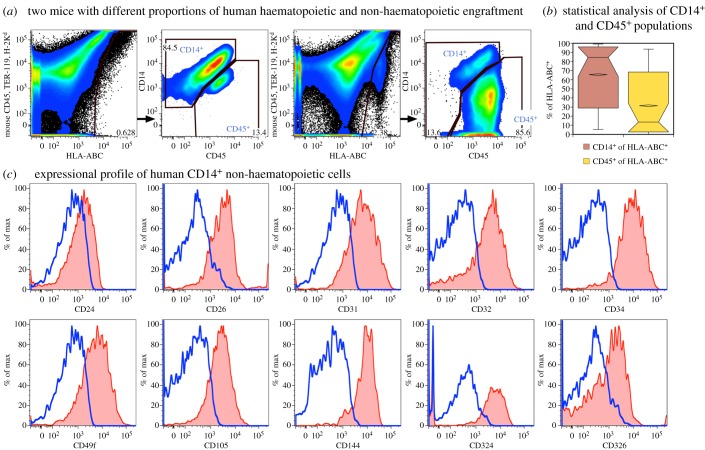
Human engraftment in mouse liver examined by flow cytometry. (*a*) Human engraftment was defined by HLA-ABC expression with reduced levels of murine CD45, TER-119 and H-2K^d^ expression. Human cells were further subdivided into CD45^+^ haematopoietic cells and CD14^+^ non-haematopoietic cells as indicated in the top row. The proportion of haematopoietic and non-haematopoietic cells varied among mice transplanted with the same cultured cells. (*b*) Notched box plots show statistical analysis of CD45^+^ and CD14^+^ cell populations (*n* = 25 mice). (*c*) Non-haematopoietic CD14^+^ human cells express both endothelial and hepatic markers.

Flow cytometric analyses of murine livers revealed two extremes of human engraftment defined by expression of CD14 or CD45 on HLA-ABC^+^ human cells, performed by a method similar to previously described [30,33]. We noted that the proportion of CD45^+^ haematopoietic to CD45^−^CD14^+^ non-haematopoietic cells varied in different mice (figure 10*a*). Descriptive statistical analysis confirmed that the non-haematopoietic population generally exceeded the haematopoietic cells in transplanted livers (*p* < 0.01, *n* = 25), but with a notable range in outcomes (figure 10*b*). Flow cytometric analyses of the non-haematopoietic cells showed that they expressed LSEC and hepatocyte markers similar to fresh and cultured fetal liver cells (figure 10*c*).

**Figure 11.**
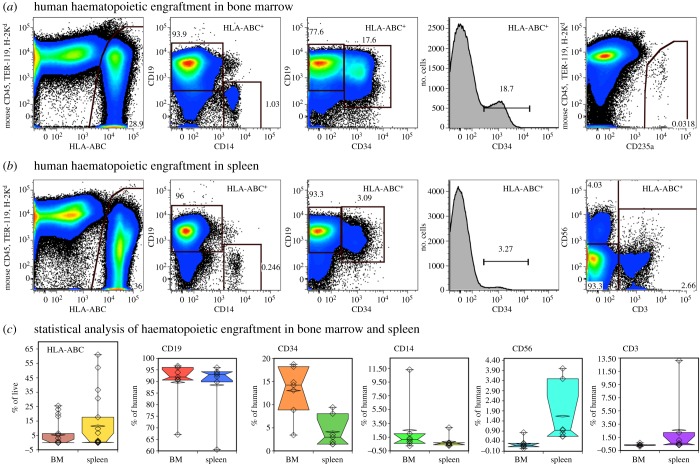
Human haematopoietic engraftment in bone marrow and spleen detected by flow cytometry. (*a*) Analysis of engraftment in the bone marrow. (*b*) Analysis of engraftment in spleen. Cells were gated for live cells and doublet discrimination was applied (not shown). CD14, CD19 and CD34 expression, as well as CD3 and CD56 in the spleen, are shown on HLA-ABC^+^ cells. (*c*) Statistical analysis of human engraftment in the bone marrow and spleen. HLA-ABC^+^ cells are shown as a percentage of live cells, *n* = 20. CD19^+^, CD34^+^, CD14^+^, CD56^+^ and CD3^+^ cells are shown as percentage of HLA-ABC^+^ cells in mice with greater than or equal to 3% engraftment (*n* = 7).

As mentioned above, cells with the phenotype of haematopoietic stem cells were preserved in liver cultures. Fresh fetal liver cells are a known source of haematopoietic stem cells capable of long-term multilineage engraftment of immunodeficient mice [41–43]. Transplantation of the cultured cells also resulted in haematopoietic engraftment in the bone marrow (figure 11*a*) and spleens (figure 11*b*). Seven to nineteen mice were examined, depending on the markers analysed, at three time points ranging from 21 to 25 weeks after transplant. The following cell populations were detected: CD14^+^ monocytes/macrophages, CD19^+^ B cells, including CD19^+^CD34^+^ B-cell progenitors, CD3^+^ T cells, CD56^+^ NK cells and CD235a^+^ erythrocytes. Statistical analysis demonstrated that human B cells were represented equally in bone marrows and spleens, whereas developing CD34^+^ cells and monocytes were more prevalent in the bone marrow (figure 11*c*). By contrast, T cells and NK cells were mostly detected in the spleen. We observed erythropoiesis only in the bone marrow because these cells are mostly lost during the isolation of light-density splenocytes. These data demonstrate multilineage engraftment of human haematopoiesis, indicating the presence of haematopoietic stem cells among the cultured cells used to engraft the mice.

**Figure 12.**
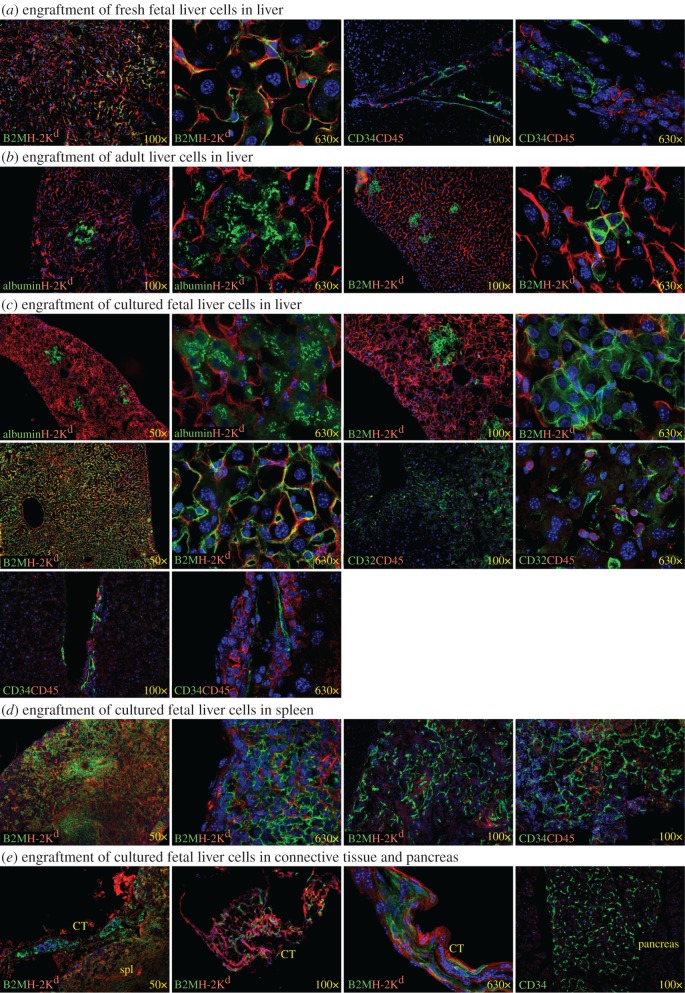
Human engraftment in TK-NOG mice. (*a*) Endothelial and haematopoietic engraftment of human fresh fetal liver cells in mouse liver. (*b*) Engraftment of adult hepatocytes in liver. (*c*) Transplantation of cultured fetal liver cells results in hepatocyte, endothelial and haematopoietic cell engraftment in liver. (*d*) Engraftment of haematopoietic and endothelial cells in spleen. (*e*) Extra-splenic engraftment of cultured fetal liver cells detected in connective tissue and pancreas. All markers are human except of mouse H-2 K^d^ and DAPI-stained nuclei are blue. CT, connective tissue; spl, spleen.

TK-NOG mice were recently described as an improved model for constructing mice with humanized livers [34]. These mice have the same immunodeficient background as uPA-NOG mice. Hepatocyte-specific ablation in TK-NOG is controlled by expression of the herpes simplex virus type 1 thymidine kinase after administration of ganciclovir. In order to compare this model with uPA-NOG mice, we transplanted TK-NOG mice with human liver cells from different sources: fresh fetal liver, adult hepatocytes and cultured fetal liver cells (figure 12). As reported previously for transplants using uPA-NOG mice [30], fresh fetal liver cells could engraft CD34^+^ endothelial and CD45^+^ haematopoietic engraftment in the TK-NOG mouse liver (figure 12*a*), whereas hepatocyte engraftment was not observed. Adult hepatocytes transplanted into TK-NOG mice, however, did yield engraftment of hepatocytes expressing albumin (figure 12*b*). Cultured fetal liver cells, on the other hand, could engraft multiple cell lineages: albumin^+^ hepatocytes, CD32^+^ LSECs and CD34^+^ vascular endothelial cells and CD45^+^ haematopoietic cells (figure 12*c*). Note, as seen under high magnification, that human albumin is concentrated close to the cell membrane borders among human hepatocytes reflecting polarization of the cells. Pan-human marker B2M helped to visualize cell morphology for hepatocytes and demonstrated abundant LSEC engraftment in some mouse livers. Human endothelial CD34^+^ and haematopoietic CD45^+^ engraftment was also detected in mouse spleens (figure 12*d*). Human endothelial cells were also found in the connective tissues around spleens and pancreases (figure 12*e*). Therefore, cultured human fetal liver cells engraft TK-NOG mice and uPA-NOG mice leading to engraftment of human hepatocytes, LSECs (and other endothelial cells) in addition to haematopoietic cells.

## 4. Discussion

In this study, we show cultures of human fetal liver are able to generate cells that can lead to hepatocyte, LSEC and haematopoietic stem cell engraftment in immunodeficient mice. Fetal liver cultures generated complex adherent cell layers composed of multiple cell types. Approximately a quarter to a third of the adherent cells were CD326^++^ hepatocytic cells, which increased in frequency over the course of the culture. These cells included albumin^+^ hepatocytes and CK19^+^ cholangiocytes, as well as hepatoblasts expressing both markers. Endothelial cells formed colonies interspersed among the other adherent cells. Small round haematopoietic cells, that were phase-bright when viewed with phase-contrast microscopy, were found to adhere to hepatocytic cells. Some larger irregular shaped haematopoietic cells also formed part of the adherent cell layer, often on the borders of endothelial cell islands. These cells most probably represented CD45^+^CD14^+^ macrophages observed in flow cytometric analyses of the cultures. Dense cell clusters were also observed in the cultures that appeared to contain a mixture of haematopoietic and hepatocytic cells, although other cell types may also be present. Thus, cultures of fetal liver cells are able to support multiple cell lineages that organize into varied cellular environments.

Our work builds on a number of reported studies that have focused on culturing specific subsets of human fetal liver cells. Lázaro *et al*. [38] described cultures that supported hepatocytic cells from human fetal liver. They reported that the hepatocytic cells were comprised of large and small cells, as we have also observed, and that expression of hepatocyte and cholangiocyte genes were maintained for six weeks in culture. Interestingly, these investigators also observed small ‘blast-like' cells that expressed CD34 and CD90, which they speculated that some might be haematopoietic stem cells, but did not perform functional analyses to verify this hypothesis. Further study by Dan *et al*. [44] of these blast-like cells in long-term cultures, maintained on fibroblast feeder layers, resulted in the isolation of a multipotent stem cell population capable of generating hepatocytes, cholangiocytes as well as mesenchymal and endothelial cell types. Fetal liver cultures have also been grown under conditions used to support haematopoiesis in long-term bone marrow cultures [45]. Stromal layers were reported to develop to confluence within a few weeks, during which time morphologically recognizable hepatocytes disappeared to be replaced by cells with the appearance of mesenchymal stromal cells. Such cultures rapidly shifted the haematopoietic output from erythropoiesis to myelopoiesis. By focusing on providing culture conditions previously shown to be supportive of CD326^++^ hepatocytic cells [28], our cultures preserved these cells while also maintaining haematopoietic and endothelial elements.

Cultured fetal liver hepatocytes demonstrated improved engraftment in uPA-NOG and TK-NOG mice. A previous study by Zhang *et al*. [29] demonstrated that transplantation of human fetal hepatocytes resulted in less engraftment than with adult cells. Our own experience with transplants performed in uPA-NOG mice yielded no hepatocyte engraftment with freshly isolated fetal cells, yet adult hepatocytes engrafted [28]. Indeed, we have achieved the highest levels of hepatocyte engraftment in uPA-NOG mice using liver cells from older donors rather than younger donors, in contrast with what we would have predicted [33]. We speculated that short-term culture of fetal cells would mature the hepatoblasts, improving their ability to engraft in an adult recipient. To stimulate hepatocyte maturation, OSM was added to the culture medium. OSM was shown to stimulate the maturation of murine [46,47] and human fetal hepatocytes [48]. As culture results in many changes to gene and protein expression, it remains to be determined what the key molecular changes are that enable better engraftment of cultured fetal hepatocytes. It is also worth emphasizing that even after culture, high levels of CD326 expression and co-expression of albumin and CK19 was observed, characteristics that define fetal hepatoblasts and not adult hepatocytes. Thus, our short-term cultures have not fully matured the fetal cells to match the phenotypic characteristics of adult hepatocytes.

Endothelial cells contribute another important aspect to modelling the fetal liver *in vitro*. Hepatocytes and LSECs develop in parallel in the fetus, providing reciprocal support for each other's development [49,50]. Experiments with rat liver cells demonstrated that interaction between hepatocytes and LSECs are important for the mutual stabilization of both types of cells, increasing albumin production, and maintaining cytochrome P1A1/2 activity by hepatocytes and retention of LSEC viability and phenotype [51]. In our cultures LSECs formed islands among hepatocytic cells with haematopoietic cells often localized on the borders of these islands. LSECs sustained in our cultures exhibited a cell-surface expressional profile similar to freshly isolated cells. The cultured LSECs maintained the capacity to engraft the livers of mice, thus *ex vivo* expansion of LSECs may prove a viable option for generating grafts to treat haemophilia A [22]. We did not supplement the cultures with vascular endothelial growth factor (VEGF) to support LSEC growth. Hwa *et al*. [52] have observed that rat hepatocytes can support the survival of LSECs without the addition of VEGF to the cultures and hepatocytes have been shown to be a source of VEGF [49]. Murine hepatocyte condition medium was also shown to support endothelial cells grown from human endothelial progenitor cells in culture [53].

Haematopoietic stem cells and progenitors were supported in the fetal liver cultures. These cells require cytokines for their survival and growth, and short-term and long-term cytokine-supported cultures of fetal liver haematopoietic stem cells have been reported that support early haematopoietic precursors [54–57]. In this study, we did not add any haematopoietic cytokines to the cultures to support stem cells or haematopoiesis in general. Indeed, OSM used in the cultures is a known inhibitor of haematopoiesis [58]. Nevertheless, a spectrum of haematopoietic precursors ranging from primitive cells expressing CD133 and high levels of CD34 to more differentiated haematopoietic precursors lacking CD133 expression and expressing low levels of CD34 were observed in the fetal liver cultures. The presence of haematopoietic stem cells in the cultures was demonstrated by the long-term multilineage engraftment in mice, indicating that cells in the mixed cultures, such as hepatocytes and endothelial cells, provide sufficient stimuli to maintain an active haematopoietic environment. Fetal liver cultures may prove useful in studying the different cellular niches that contribute to prenatal haematopoiesis.

Transplantation of uPA-NOG and TK-NOG mice with cultured fetal liver cells not only provided a means of demonstrating the viability of different cell lineages present in the cultures, but also demonstrated further advancement in our ability to construct mice with humanized livers. In this study, we demonstrated successful engraftment of three lineages: hepatocytes, haematopoietic and endothelial cells. Some recent studies have demonstrated hepatocyte engraftment along with blood cell engraftment [40,59–61]. Our group has previously detailed multilineage haematopoietic engraftment of the livers of uPA-NOG mice in addition to LSEC engraftment using freshly isolated fetal liver cells [62], but without hepatocyte engraftment [30]. Although cultured fetal liver cells have now yielded hepatocyte engraftment, the levels of engraftment using these cells are still below what others have reported using different strains of mice [63–66].

We believe that a critical aspect for supporting high-level engraftment, besides the source and age of donor cells, is the degree of liver injury in the host mice. Even modest differences in the degree of liver injury can affect engraftment levels [33]. Mice that have constitutive expression of an uPA transgene in the liver offer a continuous proliferative advantage to donor cells unaffected by the ectopic uPA expression [67]. However, the generation of such mouse strains requires a balance between mice with a severe hepatic deficiency and mice that are viable and fecund. The uPA-NOG mice used in this study were created to balance these two factors and, consequently, may not be the most permissible hosts for fetal hepatoblasts [32]. Mouse strains with an inducible liver injury, such at TK-NOG mice [34], offer the possibility to maintain healthy colonies of recipient mice that only suffer liver injury when desired. However, with a single ganciclovir treatment such mice only suffer acute liver damage rather than providing an environment that continuously favours human hepatocyte proliferation. In our comparison of two mouse strains, we observed no notable difference in engraftment between the two strains. However, hepatocyte engraftment in TK-NOG mice may be further improved with higher doses of ganciclovir treatment prior to transplant and/or repeated treatments after transplant to promote the growth of human hepatocytes.

The highly proliferative nature of fetal haematopoietic stem cells allows for the engraftment of uPA-NOG and TK-NOG mice without any prior cytoablation of the bone marrow. Previously, Gutti *et al*. [40] experimented with co-transplantation and sequential transplantation of human fetal hepatocytes and haematopoietic cells, but only haematopoietic engraftment was achieved. Only when adult hepatocyte transplants were mixed with fetal haematopoietic grafts was dual lineage engraftment achieved. Similarly, Strick-Marchand *et al*. [60] constructed humanized mice using adult hepatocytes combined with fetal haematopoietic cells. These investigators did not discern any affect of the allogeneic haematopoietic transplants on the levels of hepatocyte engraftment and vice versa. In our experiments using syngeneic cells, we also observed a high degree of variance in the levels of engraftment of different lineages, suggesting that mechanisms other than immune reactions between donor tissues are responsible for variability in engraftment. It is also worth noting that, like Strick-Marchand *et al*. [60], we observed hepatocyte polarization seen as clusters of human hepatocytes with apical accumulation of albumin. Staining showed LSEC engraftment surrounding the human hepatocytes with abundant CD45^+^ blood cells in the sinusoids, thus reconstituting major elements of human liver architecture in an animal model that may prove valuable in the study of liver pathologies and infectious diseases.

## 5. Conclusion

Human fetal liver cultures are described that support multiple cell lineages with minimal addition of exogenous growth factors or serum. Fetal hepatocytes exposed to *ex vivo* culture demonstrated improved engraftment in mice, while transplantable LSECs and haematopoietic stem cells were also maintained in the cultures. Multilineage human fetal liver cultures offer a multitude of possibilities for studying liver development and function. We see such cultures also playing an informative role in developing cell therapies requiring the generation of hepatocytes, haematopoietic stem cells and/or LSECs from pluripotent stem cells or other stem cell sources. The use of cultured fetal liver cells as graft material for constructing mice with humanized livers also offers additional possibilities for developing improved animal models to study human liver function and disease.

## Data accessibility

This article has no additional data.
